# Health care provider payment schemes and their changes since 2010 across nine Central and Eastern European countries – a comparative analysis^☆^

**DOI:** 10.1016/j.healthpol.2025.105261

**Published:** 2025-03

**Authors:** Costase Ndayishimiye, Marzena Tambor, Daiga Behmane, Antoniya Dimova, Alina Dūdele, Aleksandar Džakula, Barbora Erasti, Péter Gaál, Triin Habicht, Pavel Hroboň, Liubove Murauskienė, Tamás Palicz, Silvia Gabriela Scîntee, Lenka Šlegerová, Cristian Vladescu, Katarzyna Dubas-Jakóbczyk

**Affiliations:** a Institute of Public Health, Faculty of Health Sciences, Jagiellonian University Medical College, Krakow, Poland; b Doctoral School of Medical and Health Sciences, Jagiellonian University Medical College, Krakow, Poland; c Riga Stradiņš University, Riga, Latvia; d Faculty of Public Health, Medical University - Varna, Bulgaria; e School of Medicine, University of Zagreb, Croatia; f Department of Public Health, Institute of Health Sciences, Faculty of Medicine, Vilnius University, Lithuania; g Data-Driven Health Division of the National Laboratory for Health Security, Health Services Management Training Centre, Semmelweis University, Budapest, Hungary; h Department of Applied Social Sciences, Faculty of Technical and Human Sciences, Sapientia Hungarian University of Transylvania, Târgu-Mureș, Romania; i World Health Organization Barcelona Office for Health Systems Financing, Barcelona, Spain; j Advance Healthcare Management Institute, Prague, Czech Republic; k National Institute of Health Services Management, Bucharest, Romania; l Institute of Economic Studies, Faculty of Social Sciences, Charles University, Prague, Czech Republic; m Faculty of Medicine, University Titu Maiorescu, Romania

**Keywords:** Provider payment scheme, Provider payment method, Health care provider, Central and Eastern Europe

## Abstract

Health care provider payment schemes consist of a complex set of arrangements used to influence provider behavior towards specific health policy objectives. The study aimed at: 1) providing a structured, comparative overview of current payment schemes within the public health system in selected Central and Eastern European (CEE) countries for different health care providers; 2) identifying and comparing major changes in payment schemes since 2010. Methods included: 1) data collection form development; 2) desk research; 3) national experts’ consultations; 4) comparative analysis. The results indicate that the nine CEE countries (Bulgaria, Croatia, Czechia, Estonia, Latvia, Lithuania, Hungary, Poland, and Romania) show numerous similarities in provider payment method mix and in the general direction of the recent changes conducted in this field. Output-based payment methods prevail across all countries and types of providers. Primary health care (PHC) providers are characterized by the most diverse payment method mix. PHC and hospital inpatient care have experienced the most frequent changes in their payment schemes within the last 13 years. These focused mostly on modifying existing payment methods (e.g. detailing payment categories), and applying additional methods to pay for specific services or performance (e.g. fee-for-service, bonus payments). The objectives of conducted change were often similar, thus, there is high potential for a shared, cross-country learning.

## Highlights

• Central and Eastern European countries are increasingly using blended payment methods.
• Recent changes focused on modifying existing payment methods and/or adding new ones.
• Modification often included: detailing payment categories, changing the principles of tariff valuation.
• The same objectives were often pursued by different changes to payment methods.
• Evaluations should be part an integral part of the provider payment reforms projects.

## 1 Introduction

Central and Eastern European (CEE) countries share many common characteristics in terms of financial and organizational aspects of their health systems [[1], [2], [3]]. This is related both to the common historical background, especially health systems transformation during the post-Soviet period [1,4,5], and more recent common reform trends [[6], [7], [8]]. Currently, among the 11 CEE countries that are European Union (EU) members (Bulgaria, Czechia, Estonia, Croatia, Latvia, Lithuania, Hungary, Poland, Romania, Slovenia, Slovakia) the public health expenditures predominates, with the main role of the social health insurance schemes, though out-of-pocket payments are still high in some of the countries [9]. In most countries, a single, centralized public payer operates. CEE countries often face similar structural challenges within their health systems, including e.g. overcapacities in hospital care [7] with simultaneous deficits in long term-care provision (LTC) [10] and fragmented primary health care (PHC) [6]. Many recent common health reform trends focused on these challenges and included diverse efforts to improve hospital governance [7,11], strengthen PHC provision and implement coordinated and/or integrated care provision models [6,8,12]. Important elements of these reforms were changes to health care provider payment schemes.

The generic objective of health care provider payment schemes is to compensate providers for the services rendered. They consist of a complex and multidimensional set of arrangements. Payment schemes include payment methods (mechanisms for transferring funds from payers to health care providers) together with all supporting elements such as contracting rules, management information systems, as well as administrative and liability mechanisms [13]. A diversity of provider payment methods exists, and each of them has its own strengths and weaknesses in different contexts [13,14]. To counter the weaknesses of single methods, policymakers pursue an optimal payment method mix [15] that must be strategically aligned with the remaining payment system elements as well as other health system capacities and objectives [14]. Depending on its design, the payment scheme creates a set of specific incentives that can influence provider behavior towards the realization of pre-defined policy objectives (e.g. more efficient use of resources, enhancing the provision of specified services, better quality of care, or responsiveness to patient's needs) [14].

There are some previous studies that include elements of comparison of provider payment schemes and their reforms in different CEE countries [7,[16], [17], [18], [19]]. However, these focused exclusively on specific sectors, e.g. hospitals [7,18], pharmaceuticals [17] or specific situations, e.g. payment adjustments during COVID-19 pandemics [19]. The **objectives of the present study** were: 1) to provide a structured, comparative overview of current payment schemes within the public health system in selected CEE countries for different health care providers; 2) to identify and compare major changes conducted in this field since 2010. The study focuses on arrangements between public payers and providers’ institutions (legal entities) and excludes issues related to the employment and remuneration of individual medical workers by health care institutions. Following the general objectives of cross-country comparative studies, we aimed at mapping relevant, recent evidence for future health care provider payment reforms in CEE countries, to indicate common trends and potential areas for shared learning and future collaborative studies.

## 2 Material and methods

The methods used comprised four consecutive phases: 1) data collection form was developed based on the literature; 2) desk research was conducted to map existing provider payment schemes and recent payment reforms in 11 CEE countries (EU members); 3) national health policy experts were identified and asked to validate and update the findings of the desk research; 4) a comparative analysis was performed.

### 2.1 Data collection form

The literature provides examples of theoretical frameworks for both the classification of provider payment methods [13,16,20], and provider payment reforms [16]. For the former, inspired by the Langenbrunner et al. [13], we distinguished between payment methods based on: 1) inputs – available or used; 2) outputs – referring to the services provided; and 3) outcomes - rewarding or penalizing based on health outcomes. Within output-based category, we further distinguished between payment per capita, per case, and per unit of service (Table 1).

**Table 1:** Payment methods classification overview.

Category	Method examples
Input-based	• Line-item budget e.g. fixed payments for salaries, global budget based on inputs
Output-based	• Per capita e.g. capitation in PHC • Per case e.g. diagnostic-related groups (DRGs), other case-based payments, pay for performance (P4P) elements when indicators include cases treated items; global budget based on cases treated • Per unit of service e.g. fee-for-service (FFS), per diem, per visit, P4P elements when indicators include services provided items, global budget based on units of services provided
Outcome-based	• P4P elements when indicators include health outcomes

Source: Based on the work of Langenbrunner et al. [13].

For mapping the provider payment method reforms, we developed a simple classification that distinguishes between three types of changes: 1) replacing the existing payment method with a new one (‘REP’); 2) modifying the existing method (e.g. by changing the number or content of reporting/coding groups, changing tariff valuation rules, changing the scope of services covered by a given method) (‘MOD’); 3) adding a new (additional) method to the existing one (‘ADD’). We also distinguished between direct changes to the payment method and all related and/or complementary changes within the payment systems, including, for example, changes in the payer structure and contracting principles (e.g., contract timeframe, volume limits).

The data collection form was developed in accordance with the two pre-defined research questions. The first part focused on the current (as of May 2023) payment schemes for the four types of care providers: 1) PHC (general practitioners (GPs)/ family doctors practices including other medical professionals working within the practice); 2) specialized ambulatory care (focused on providers with medical specialties outside general practice); 3) hospitals (focused on curative care) and 4) LTC provided within health care sector (focused on care for people dependent on an extended period of time, including e.g. nursing and palliative care). For both hospitals and LTC providers, we have distinguished between outpatient services (including day and home care) and inpatient care (patient stay minimum 24 h). When possible, we aimed at indicating the dominant payment method for each provider type, i.e. one that covers the majority of providers or services or the value of the public payer budget. The second part aimed to map the major changes since 2010 (they were listed in a chronological order according to the four types of providers). We excluded COVID-19 related payment solutions/reforms that did not continue after the pandemic. Our study focused on payments for health services (we excluded capital investments). We also excluded payments for pharmaceuticals, medical products and dental services.

### 2.2 Desk research

A desk research of standardized data sources was conducted between March and May 2023. The objective was to complete the data collection forms for the 11 CEE countries (EU members). The sources included the following report series: Health System Reviews and Health Systems Summaries [21], Health System and Policy Monitor (HSPM) [22] and Country Health Profiles - State of Health in the EU, available through the European Observatory on Health System and Policies website [23]. These reports are based on standardized methodologies, have a publication format that allows cross-country comparisons, are regularly updated and available for all EU Member States. In addition, basic indicators characterizing health system funding and providers capacities in the analysed countries were retrieved from the Eurostat database [9].

### 2.3 National expert consultations

The partially completed data collection forms for each country were sent to the national experts selected on the basis of purposive sampling. We contacted the national representatives of the Health System and Policy Monitor Network [24], the vast majority of whom are also the authors of the reports screened during the desk research. These experts have in-depth knowledge of the organization and policy processes in their national health systems [24]. Each expert was invited to participate in the study or to indicate another national informant with relevant expertise (snowballing method). The national experts were asked to validate and update the findings of the desk research and to provide appropriate references if needed. The experts have been included as co-authors of this paper.

### 2.4 Comparative analysis

The validated and updated data collection forms were analysed by two researchers (CN and KDJ) in August 2023. Dedicated classifications (see Section 2.1.) were used for both the current payment methods and their reforms. The draft comparative results were shared with all national experts, with a request for further validation and clarification. Additional questions and ambiguities were clarified iteratively through further correspondence.

## 3 Results

During the desk research, basic data were collected for all 11 CEE countries (EU members). However, much more relevant information was available for some of the countries than for the others. Also, not all national experts responded to our invitation to participate in the study and/or provided adequate inputs. Consequently, nine countries were included in the final analysis of results: Bulgaria, Croatia, Czechia, Estonia, Latvia, Lithuania, Hungary, Poland and Romania.

### 3.1 General comparison of national health systems

Table 2 presents some general characteristics of the health systems of the nine countries in relation to the funding model and providers’ capacities. In the majority of the included countries, an insurance-based health care funding model dominates with a single, centralized public payer. Latvia is the only country with a tax-based system, whereas Czechia is the only country with a multiple public payer's structure. In the case of the latter, although multiple payers exists, there is limited competition between them, and they all follow the same reimbursement regulation issued by the Ministry of Health [25].

**Table 2:** Chosen health system characteristics of the analysed countries.

Feature	Bulgaria	Croatia	Czechia	Estonia	Hungary	Latvia	Lithuania	Poland	Romania
Dominant funding model	insurance based	insurance based	insurance based	insurance based	insurance based	tax based	insurance based	insurance based	insurance based
Main public payer structure	single payer, centralized (with 28 regional branches)	single payer, centralized	multiple payers, with limited competition	single payer, centralized	single payer, centralized	single payer, centralized	single payer, centralized	single payer, centralized (with 16 regional branches)	single payer, centralized
Total CHE as a share of GDP* (2021)	8.62 %	8.01 %	9.49 %	7.54 %	7.38 %	9.11 %	7.76 %	6.44 %	6.47 %
Share of public expenditures⁎⁎ in total CHE (2021)	64.95 %	85.50 %	86.42 %	76.12 %	72.45 %	69.47 %	68.78 %	72.46 %	78.33 %
Share of the main public payer/s expenditure⁎⁎⁎ in total CHE (2021)	49.14 %	76.07 %	70.97 %	63.41 %	57.93 %	69.47 %	56.62 %	57.33 %	60.11 %
Rate of generalist medical practitioners per 100,000 pop. (2021)	59.67	82.17	71.82	233.31	66.82	76.84	102.50	88.51	79.21
Rate of specialist medical practitioners per 100,000 pop. (2021)	396.87	288.94	337.43	256.36	262.09	258.96	344.97	255.61	271.63
Rate of available hospital beds per 100,000 pop. (2021)	792.28	567.54	665.51	439.44	678.56	516.43	605.43	627.18	720.56
Rate of available beds in nursing and other residential LTC facilities per 100,000 pop. (2021)	28.81	233.96	716.98	259.33	866.86	259.33	766.31	201.40	211.92

⁎ CHE – current health expenditures, GDP – gross domestic product.

⁎⁎ government schemes and compulsory contributory health care financing schemes (SHA 2011 – HF1).

⁎⁎⁎ main financing scheme (SHA 2011 – government scheme (HF11) for Latvia and compulsory contributory health insurance (HF12) for remaining countries). Source: based on: Country Health Profiles 2021 and Eurostat database (2024).

Regardless of the funding model, in all nine countries, the share of total current health expenditure (CHE) in gross domestic product in 2021 was below the EU-27 average of 10.88 %. In the same year, the share of public expenditures in total CHE ranged from 64.95 % in Bulgaria to 86.42 % in Czechia, with the EU-27 average of 81.19 %. Public payers play a dominant role in purchasing health services in the vast majority of countries. In 2021, Bulgaria was the only country where the share of the social health insurance payer expenditure in total CHE was slightly below the EU-27 average of 50.69 % (i.e. 49.14 % for Bulgaria). The values of indicators characterizing providers’ capacities vary significantly between countries. For example, in 2021, the number of generalist medical practitioners per 100,000 inhabitants ranged from 59.67 in Bulgaria to 233.31 in Estonia. Majority of countries seems to have overcapacities in hospital care. In 2021, the number of available hospital beds per 100,000 inhabitants was above the EU-27 average of 524.76 in seven countries. At the same time, there was high diversity in the availability of LTC beds per 100,000 inhabitants, from only 28.81 in Bulgaria to 866.86 in Hungary (Table 2).

### 3.2 Mapping the current provider payment methods

Table 3 presents the results of mapping current payment methods for each provider type while Supplementary Table S1 shows these methods classification overview.

**Table 3:** Payment methods* valid as of May 2023, per country and provider type.

Type of care & provider / Country	Primary health care	Outpatient specialized care (outside hospitals)	Hospitals		Long term care (within health sector)	
			Outpatient specialized care (inside hospitals)	Inpatient hospital care	Inpatient LTC	Outpatient/day/ home LTC
**Bulgaria**	Capitation + FFS, P4P	FFS	Case payment	Case payment	Case payments (for acute episodes), Per diem (for psychiatric care)	Not covered
**Croatia**	Capitation + FFS, P4P, Fixed payment,	FFS	FFS, Case payments	Global budget, DRGs, Case payment	Per diem	FFS
**Czechia**	Capitation + FFS, P4P, Fixed payments (for emergency care shifts)	FFS + case payments (day surgeries), P4P elements (dialysis providers)	FFS + Case payments (day surgeries), P4P elements (dialysis providers)	Global budget (based on DRGs), DRGs, Fixed payment (for palliative care)	Per diem	FFS
**Estonia**	Capitation, FFS, P4P, Fixed payments (e.g. for distance, second nurse)	FFS + P4P elements (video consultations)	FFS + P4P elements (video consultations)	DRGs, FFS, Per diem, Fixed payments, Bundled payments (for stroke patients)	Per diem, FFS	FFS
**Hungary**	Capitation, Case payment, P4P, Fixed payments (for salaries and group practices)	FFS + Fixed payment (for salaries)	FFS + Fixed payment (for salaries)	DRGs + Fixed payment (for salaries)	Per diem + Fixed payment (for salaries)	Per diem (hospice home care), Per visit (specialist home care)
**Latvia**	Capitation, FFS, Fixed payment (for salaries), P4P	FFS, Case payment, Fixed payment (for salaries)	FFS, Case payment	DRGs, Case payment, Per diem, Fixed payment (for emergency care), FFS	Fixed budget, Per diem	Case payment, FFS,
**Lithuania**	Capitation, FFS, P4P, Fixed payment (for special needs patients)	Case payment	Case payment, FFS (for expensive procedures and examinations)	DRGs, FFS	Per diem, Case payment (palliative care)	Per diem, FFS, Fixed payment (nursing at home), Case payment (palliative care)
**Poland**	Capitation + Per visit/consultation, FFS (for diagnostic tests and within coordinated care), Fixed payment (for rural/low density population)	Per visit payment (groups adjusted for number and type of services provided) + FFS	Per visit payments (groups adjusted for number and type of services provided) + FFS, P4P elements (oncological network)	Global budget (based on DRGs) for hospital included in network + DRGs, P4P elements (for stroke patients), FFS, per diem	Per diem (differentiated based on health and care needs)	Per diem (differentiated based on health and care needs)
**Romania**	FFS + Capitation, P4P, Fixed payment (for newcomers)	FFS	FFS	DRGs + Case payment, Fixed payment (for salaries)	Per diem	FFS, Case payment

⁎ If a dominant payment method exists (one that covers the majority of providers or services or the value of the public payer budget) it is listed first following by other, additionally used methods after ‘+’ sign. If not dominant payment method can be defined, all methods applied for a given type of provider were listed.

#### 3.2.1 Primary health care

Primary healthcare providers are characterized by the most diverse payment method mix. In some countries, the methods used include those from all classification categories: 1) input based, 2) output based per capita, per case and per service, and 3) outcome based (Supplementary Table S1). Two common methods are capitation and fee-for-service (FFS). In most countries, these two methods are used together, and their proportions can vary. For example, Czech GPs are paid based on a combination of capitation and FFS with the: share of each method at 63 % and 37 %, respectively [26], while in Romania, the proportions are: 35 % and 65 %, respectively. In the majority of countries, these are supplemented by diverse P4P programs covering both output- and outcome-based indicators, with the prevalence of the former (see next paragraph). Some countries also use input-based methods. These include for example different forms of fixed payments for salaries in Hungary and Latvia, office maintenance expenses in Croatia, and payments for newcomers (for establishing a new practice) in Romania or distance care provision in Estonia.

In relation to P4P programs within the PHC, financial rewards can take a different form. For example, in Czechia, PHC physicians receive a higher capitation payment if they perform a complex examination of at least 30 % of the registered patients aged 40–80 years [25]. In Bulgaria, higher FFS rates are offered to PHC units that report a higher proportion of adult patients (above 60 %) covered by prophylactic examinations [27]. In Romania, an additional fixed payment is offered for achieving defined yearly target of the number of patients assessed for particular health risks. In Croatia, the bonus payment includes i.a. a fixed payment for salaries and office maintenance as well as increased capitation [28]. The indicators used within P4P in PHC can be calculated differently and apply different thresholds, however, patterns are similar across countries. In general, in many existing P4P programs, the measured indicators often relate to: 1) vaccination rates (e.g., the share of elderly population vaccinated against influenza in Hungary and Lithuania or children immunization rates in Latvia and Estonia); 2) cancer screening intensity (e.g., three pre-defined cancer types early diagnosis indicators in Lithuania or mammography rate in Hungary); and 3) chronic disease monitoring (e.g., the share of patients with hypertension, diabetes, and asthma tested for predefined clinical indicators in Latvia or indicators related to correct medicine prescriptions in chronic illnesses in Estonia and Hungary). For the latter dimension, some countries also apply outcome-based indicators. For example, in patients with diabetes mellitus, the level of glycosylated hemoglobin is monitored (with the rate set at equal or below 7 % on two occasions during the reporting period in both Hungary and Lithuania, and below 7.5 % in 60 % of patients in Latvia). Also, in relation to the chronic disease management dimension, some countries apply indicators that can be classified as both output- and outcome-based, e.g. the share of avoidable hospital admissions for a group of pre-defined chronic conditions in Croatia and Lithuania.

#### 3.2.2 Outpatient specialized care

In all analysed countries, specialized outpatient care relies on output-based payment methods, mostly FFS and case payments (Table 3). In most countries, there are no major differences in payment methods for services provided outside or within hospital settings. FFS is used in all countries. In three countries, elements of P4P are used for: video consultations in Estonia, dialysis providers in Czechia, and meeting diagnostics time targets for oncological patients in Poland. In Hungary and Latvia FFS is supplemented by an additional fixed salary budget.

#### 3.2.3 Hospital inpatient care

Diagnostic related groups (DRGs) are the most commonly used payment method for hospital inpatient care. Bulgaria is the only country without a dedicated DRGs scheme – case payments based on clinical pathways are used instead [29]. In most of the remaining eight countries, DRGs are supplemented by other output-based payments (e.g., per diem, FFS) and/or fixed payments (Table 3 and Supplementary Table S1). In the case of the latter, for example in both Romania and Hungary fixed payments for salaries are used. In Hungary, they constitute approximately 40 % of the total income of providers [30]. In Czechia, a fixed payment is used for palliative care provision in hospitals (calculated based on number of insured population) [31], while in Latvia it covers emergency care (calculated based on the number of emergency care doctors). In Poland, P4P elements are used within the coordinated care model for patients with acute myocardial infarction. Hospitals that decide to participate receive financial bonuses which take the form of an increased refund from the payer (depending on the share of patients included in the program) or an additional bonus payment for each patient who is able to return to work within four months of discharge from the hospital [32,33]. Estonia also introduced a coordinated care program for stroke patients, with bundled payments covering the entire course of treatment and related complications for a period of 365 days. The covered services include acute treatment, rehabilitation, follow-up visits, and nursing care [34].

#### 3.2.4 Long-term care

In the case of long-term care services provided within the health care system the vast majority of countries rely on simple output-based methods, mostly per diem and FFS (Table 3). Bulgaria is the only country, where outpatient LTC is not exclusively covered by public payer. All nine countries use per diem for LTC inpatient care (either as a solo method or in combination with: case payment in Bulgaria and Lithuania, FFS in Estonia and fixed payments in Hungary and Latvia). The same type of methods is common for LTC outpatient, day and home care. In general, the specific LTC payment methods are often chosen to the specific scope of services and disease categories. For example, in Latvia, home palliative care for oncological patients is covered by case payments, while inpatient care for patients with mental disorders is financed via fixed budgets.

### 3.3 Changes in provider payment schemes since 2010

Table 4 presents an overview of the main changes in payment methods conducted since 2010 per country and provider type (Supplementary Table S2 presents extended version of this table, with description of the main motivations behind each change). Table 5 shows a clustered overview of the motivations behind the changes in payment methods, while Table 6 lists the major changes to other payment system's elements.

**Table 4:** Overview of main changes to payment methods*, per country and provider type, since 2010.

Type of care & provider / Country	Primary health care	Outpatient specialized care (outside hospitals)	Hospitals		Long term care (within health sector)	
			Outpatient specialized care (inside hospitals)	Inpatient hospital care	Inpatient LTC	Outpatient/day/ home LTC
**Bulgaria**	**‘MOD’** (ongoing: new capitation tariffs, age adjusted FFS, FFS tariff modification) + **‘ADD’** (2022: P4P elements for prophylaxis)	**‘MOD’** (new tariffs valuation)	**‘MOD’** (2016: new ambulatory procedures, new tariffs)	**‘MOD’** (increased no of clinical pathways)	‘NO CHANGES’	‘NO CHANGES’
**Croatia**	**‘ADD’** (2013: P4P)	‘NO CHANGES’	**‘MOD’** (2015: introducing diagnostic procedures)	**‘MOD’** (2015: refined DRGs)	‘NO CHANGES’	‘NO CHANGES’
**Czechia**	**‘MOD’** (ongoing: increasing capitation, expanding the scope of services financed via FFS) + **‘ADD’** (2016: fixed bonus/payment for emergency care shifts)	**‘MOD’** (ongoing: new FFS tariffs, expanding the scope of services financed via FFS) + **‘ADD’** (2019–2020: P4P for dialyses providers, 2023: case payment for day surgery)	**‘MOD’** (ongoing: changes in reimbursement formula, expanding the scope of services financed via FFS) + ‘**ADD’** (2019–2020: P4P for dialyses providers, 2023: case payment for day surgery)	**‘MOD’** (2012: activity based global budgets, 2019: DRGs modifications) + **‘ADD’** (2023: fixed payment based on number of insured for palliative care) + **‘REP’** (2021: CZ-DRGs)	**‘MOD’** (2016: ongoing tariffs differentiation, new reimbursement rules)	**‘MOD’** (2018–2019: expanding scope of services financed via FFS)
**Estonia**	**‘MOD’** (2012: new capitation groups, ongoing: tariff valuation changes) + **‘ADD’** (2013: FFS for e-consultations; 2015: P4P, 2017: fixed payment for group practice)	**‘ADD’** (2021: video consultations P4P)	**‘ADD’** (2021: video consultations P4P)	**‘ADD’** (2020: fixed payment for emergency care; 2021: bundled payment for stroke patients)	**‘MOD’** (ongoing tariffs differentiation)	**‘MOD’** (ongoing tariffs differentiation)
**Hungary**	**‘ADD’** (2021: fixed payment to encourage group practices, 2021: fixed payment for salaries)	**‘ADD’**(2021: fixed payment for salaries)	**‘ADD’** (2021: fixed payment for salaries)	**‘MOD’** (ongoing tariffs adjustments) + **‘ADD’** (2021: fixed payment for salaries)	**‘ADD’**(2021: fixed payment for salaries)	‘NO CHANGES’
**Latvia**	**‘ADD’** (2013: FFS and P4P)	**‘MOD’** (ongoing tariffs adjustments)	**‘MOD’** (ongoing tariffs adjustments)	**‘REP’** (2011: DRGs) + **‘MOD’** (DRGs modifications)	**‘MOD’** (ongoing: changes in the scope of services financed via given method)	**‘MOD’** (ongoing: changes in the scope of services financed via given method)
**Lithuania**	**‘MOD’** (expanding the scope of services financed via FFS and P4P)	**‘MOD’**(new tariffs, expanding the list of services financed via case payments; 2016: extended consultations)	**‘MOD’**(new tariffs, expanding the list of services financed via given methods; 2016: extended consultations)	**‘REP’** (2012: DRGs) + **‘MOD’** (2015: country specific DRGs weights)	**‘MOD’** (2018–2022: ongoing tariffs differentiation)	**‘MOD’** (2019: new tariffs for palliative care)
**Poland**	**‘MOD’** (adjusted capitation groups, expanding scope of services financed via FFS) + **‘ADD’** (2019: fixed payment for rural areas)	**‘REP’** (2011: per visit) + **’MOD’** (new tariff valuation rules, new rules for reimbursement calculation)	**‘REP’** (2011: per visit) + **‘MOD’**(new tariff valuation rules, new rules for reimbursement calculation) + **‘ADD’**(2017–2022: global budget, 2015: P4P elements within oncological network, FFS for oncological coordinated care)	**‘MOD’** (new tariff valuation rules, new rules for reimbursement calculation) + **‘ADD’** (2017: global budget; 2015: P4P elements within oncological pathways, FFS and per diem for oncological coordinated care, 2022: P4P elements within stroke program)	**‘MOD’**(2015: gradual tariffication of services, differentiation of per diem payment depending on health needs)	**‘MOD’**(2015: gradual tariffication of services; differentiation of per diem depending on health needs)
**Romania**	**‘MOD’** (expanding the scope of services financed via FFS) + **‘ADD’** (2023: P4P)	**‘MOD’** (ongoing changes to the tariff valuation rules)	**‘MOD’** (extending the list of services financed via FFS, ongoing changes to the tariff valuation rules)	**‘MOD’** (new tariff rules for DRGs in Romanian context 2020) + **‘ADD’** (2017: additional, fixed payment for salaries increase)	‘NO CHANGES’	**‘MOD’** (2014: changes to tariffs calculation methods for home care) + **‘ADD’**(2018: payment for outpatient palliative care)

⁎ Major changes to payment methods, including four options: ‘NO CHANGES’ (no change in the payment methods since 2010 as well as no major modification to its content); REP’ – replacing previous method to new one; ‘MOD’ – modifying the existing method (e.g. by changing number of DRGs/capitation groups, modifying costing groups by making them more detailed, changing tariff valuation rules, expanding the scope of services financed via given method); ‘ADD’ – adding new (additional method) to the existing one.

**Table 5:** Overview of the main motivations behind payment methods changes*, per provider type, since 2010.

Type of care & provider		Motivations → examples of payment method changes
**PRIMARY HEALTH CARE**		• Better reflection of actual costs → MOD: age adjusted capitation and FFS; capitation and FFS tariffs increase • Expanding the scope of PHC services → MOD: capitation/FFS tariffs increase; expanding scope of services financed via FFS; ADD: P4P • Enhancing disease prevention activities → MOD: expanding scope of services financed via FFS; expanding scope of services included in P4P; ADD: P4P • Encouraging setting up group/multidisciplinary practices → ADD: fixed payment • Encouraging provision of services for rural populations → ADD: fixed payment • Encouraging PHC doctors to work in emergency care → ADD: fixed payment • Better care coordination (for chronic conditions) → MOD: expanding scope of services financed via FFS; ADD: P4P • Covering regulatory wages increase → ADD: fixed payment
**OUTPATIENT SPECIALIZED CARE (outside hospitals)**		• Better reflection of actual costs → MOD: FFS and case payment tariffs’ modification; REP: introduction of adjusted per visit payments • Encouraging more services being provided in outpatient settings → MOD: expanding scope of services financed via FFS and/or case payments and updating their tariffs, ADD: P4P, case payment • Improving access and quality of care → ADD: P4P • Covering regulatory wages increase → ADD: fixed payment
**HOSPITALS**	**Outpatient specialized care (inside hospitals)**	• Better reflection of actual costs → MOD: FFS and Case payment tariffs modifications; REP: introduction of adjusted per visit payments • Encouraging more services being provided in outpatient settings → MOD: expanding scope of services financed via FFS and/or case payments and updating their tariffs; ADD: P4P, case payment, global budget • Improving access and quality of care → ADD: P4P • Covering regulatory wages increase → ADD: fixed payment • Better care coordination: ADD → P4P, FFS, bundled payment
	**Inpatient hospital care**	• Better reflection of actual costs → MOD: DRGs’ and tariffs’ modification, case payments groups and tariffs’ modification • More effective resources use → REP: introducing DRGs; MOD: DRGs’ modification • Better flexibility of the types of services provided → MOD: activity-based budgets; ADD: global budget • Better care coordination: ADD → P4P, FFS, bundled payment • Encouraging provision of specific services → ADD: fixed payment • Covering regulatory wages increase → ADD: fixed payment; MOD: adjusting tariffs valuation
**LONG TERM CARE (within health sector)**	**Inpatient LTC**	• Better reflection of actual costs → MOD: FFS, per diem, case payment tariffs’ modification • Covering regulatory wages increase → ADD: fixed payment
	**Outpatient/day/ home LTC**	• Better reflection of actual costs → MOD: FFS, per diem, case payments tariffs modifications • Encouraging more services being provided in outpatient/day/home settings → MOD: expanding the scope of services financed via activity-based payments (FFS, per diem, case payments); ADD: case payments

⁎ ’REP’ – replacing previous method with the new one; ‘MOD’ – modifying the existing method; ‘ADD’ – adding new (additional method) to the existing one.

**Table 6:** Overview of additional main changes within the payment system, per provider type, since 2010.

Primary health care	Outpatient specialized care (outside hospitals)	Hospitals		Long term care (within health sector)	
		Outpatient specialized care (inside hospitals)	Inpatient hospital care	Inpatient LTC	Outpatient/day/ home LTC
• new contract rules to support multidisciplinary care/ group practices (Estonia, Hungary) or coordinated care (Poland) • new rules for P4P (Lithuania)	• ongoing changes to volume limits settings (Estonia, Hungary, Poland) • capped total reimbursement (since 2016 in Czechia)	• ongoing changes to volume limits settings (Estonia, Hungary, Poland) • expanding contracts timeframe (Lithuania, Poland) • new administration system, shifting from fragmented to comprehensive contracts (Lithuania)	• ongoing changes to volume limits settings (Estonia, Hungary, Poland); caps on admissions (Bulgaria) or expenditure (Croatia) • expanding contracts timeframe (Estonia, Lithuania, Poland) • new administration system, shifting from fragmented to comprehensive contracts (Lithuania)	• no major changes	• ongoing changes to volume limits to home care (Romania)

#### 3.3.1 Primary health care

In the case of primary care providers, the majority of countries modified existing payment methods and/or added new (additional) ones (Table 4). In the former case, changes often focused on adjusting costing items (e.g. capitation and FFS) and changing tariffs valuation rules to better reflect actual costs. Increasing tariffs valuation as well as expanding the scope of services financed via FFS usually aimed at encouraging more PHC services provision. Six countries added P4P schemes while in two countries, these were already in place prior to 2010. P4P programs often focused on enhancing disease prevention activities and/or improving care coordination (for chronic conditions). These two objectives were also pursued by expanding the list of services financed via FFS (e.g. in Poland). Several countries introduced an additional fixed payment, yet often with different objectives (e.g. Poland to encourage the provision of services for rural/low population density areas; Hungary and Estonia to encourage the establishment of group GPs practices; Czechia to encourage PHC doctors to take emergency department shifts) (Table 5 and Supplementary Table S2).

#### 3.3.2 Outpatient specialized care

The main motivations behind the changes in payment methods for outpatient specialized care (provided both in- and outside hospitals) were focused on better reflection of actual costs and encouraging more services being provided in outpatient settings (Table 5 and Supplementary Table S2). The majority of countries modified the existing payment schemes by adjusting tariffs valuation rules and/or expanding the list of procedures provided in outpatient or daycare settings. Some countries added new payment methods. For example, in 2021, Hungary introduced an additional fixed payment for salary increases, whereas Estonia introduced additional performance payments (within P4P program) for video consultations. In both countries, additional payments covered all publicly funded outpatient specialist visits, both outside and inside hospitals. Improving access and quality of care was pursued by implementing P4P elements in Czechia (for dialyses providers) and Poland (within the oncological network). In the case of outpatient specialized care provided in hospitals, the objective of better care coordination was pursued by introducing bundled payments for stroke patients in Estonia, P4P elements and new FFS for oncological and stroke patients in Poland (Table 4, Table 5). Changes in payment methods were complemented by changes to other payment scheme principles (Table 6). For example, Estonia, Hungary, and Poland changed the rules for calculating the volume limits that covered outpatient services provided both inside and outside hospitals. In Poland, volume caps were initially removed in 2019 for selected ambulatory procedures, while from 2021, volume caps for both specialist consultations and diagnostic procedures were completely removed with the objective of encouraging service provision in the outpatient setting and mitigating the problem of long waiting times. In Hungary, the methods for calculating output volume limits for specialist care providers have been changed several times (in 2011, 2014, and 2021). From 2023 onwards, the following parameters are used to plan the annual performance limit: the historical performance in two years prior to the COVID-19 outbreak, the health needs of the local population, and the structure of medical specialties in the hospital.

#### 3.3.3 Hospital inpatient care

Payment schemes for hospital inpatient care appeared to have undergone the most substantial reforms since 2010. Three countries replaced the dominant method: Latvia introduced DRGs (based on the Nordic system) in 2011, Lithuania a year later (based on the Australian system), while Czechia implemented its new version ‘CZ-DRG’ (under the DRG restart program) in 2021 (Table 4). In all three countries, the main objective was to improve the efficiency of hospitals (more effective use of resources) and transparency of the payment scheme. The majority of countries modified the existing payment method by expanding and/or changing the reporting group categories (e.g., by making them more detailed) with the objective of assuring better reflection of the actual costs of services. Also, most countries made efforts to improve tariff valuation methodology (e.g., by changing the DRGs costing base and/or adjusting tariffs to the national/local context). For example, in Romania, these were preceded by dedicated research projects [35], whereas in Poland, a dedicated Tariffication Agency was launched in 2015 [36]. The issue of increasing salaries for medical staff had a strong impact on the changes in payment schemes in some countries. In Poland, the new central regulation on annual increases in medical staff wages was passed in 2017 [37], but only since 2022 these wage increases have been included in the new tariff valuation methodology. In Romania and Hungary, additional fixed payments for salary increases were introduced (in 2017 and 2021, respectively). The objectives of greater flexibility in the types of services provided was pursued by modifying (in Czechia) or introducing (in Poland) hospital global budgets, while better care coordination (especially with out-patient care) was pursued by bundled payments in Estonia and P4P elements and new FFS for oncological and stroke patients in Poland (Table 5).

Changes in payment methods for inpatient hospital care were often accompanied by changes to other payment principles (Table 6). In both Poland and Lithuania, the timeframe of hospital contracts with public payers was extended (from one year to four and three years respectively). In Estonia, the contract period is five years (and the previously existing timeframe difference between private and public hospitals was eliminated). In Poland, the change was part of the broader hospital network reform implemented in 2017, which aimed to improve the organization of hospital services [38]. In Lithuania, the shift from annual to three-year contracts was accompanied by updating the rules for contracting procedures. In most countries, the issue of hospital care volume limits was an important element of the reforms, often focused on limiting hospital expenditures and/or more effective resources’ use. In Bulgaria, for example, there were formal ceilings on hospital admissions per hospital and for each clinical care pathway between 2015 and 2018, while in 2018, a ban was introduced for public payer contracting with new hospitals and for new hospital activities [39]. In Croatia, a ceiling for hospital expenditure has existed since 2015 (hospitals started to be paid upfront and report subsequent care episodes) [28]. In Poland, one of the indirect objectives of the 2017 hospital network reform was to eliminate the problem of hospitals providing services above their contracted limits. Under the new payment model (global budget), a hospital can only receive limited reimbursement for services provided above the budget value on the condition that other hospitals in the region have not utilized their budgets [38]. Similarly in Hungary, a degressive zones were introduced in 2010 with the aim of more efficient resources’ allocation and improved access to care (services provided above the limits were reimbursed, yet covering only part of their costs).

#### 3.3.4 Long-term care

Changes in payment schemes for long-term care services (provided in the health sector) were the least common. Countries that introduced changes often focused on modifying or recalibrating tariffs aimed at better reflection of actual costs and/or expanding the scope of services financed via given method, often to incentivize the provision of LTC services in out-patient, day or home settings.

## 4 Discussion

Our study shows numerous similarities between the nine CEE countries, both in terms of current payment methods per provider type and the main changes implemented in this field since 2010. Capitation is used for paying PHC providers in all nine countries, yet constitutes the dominant method in only four of them. For the remaining providers, output-based payment methods, calculated per unit of service (e.g. FFS, per diem) and per case treated (e.g. case payment) prevail across all countries (Table 3 and Supplementary Table S1). Payments based on available inputs usually take the form of dedicated fixed payments (e.g., for salaries), while outcome-based methods can be partially embedded into existing pay-for-performance programs in PHC (when reported indicators refer to health status).

All nine countries have implemented changes within healthcare provider payment schemes since 2010. Reforms seemed to occur more frequently in PHC and hospital inpatient care than in the remaining types of providers. The most frequently conducted changes to payment methods focused on modifying existing methods (‘MOD’), followed by adding a new (additional) method to the existing ones (‘ADD’) (Table 4). The main motivations behind conducted changes were often similar, including e.g. better reflection of actual costs (common objective across all provider types); expanding the scope of PHC services (often focusing on disease prevention, care coordination, and multidisciplinary care); shifting emphasis from in- to outpatient care provision (for both hospitals and LTC units), improved efficiency of hospital care (Table 5). In some countries, changes to payment methods were accompanied by changes to other elements of the payment scheme. These often included: introducing, changing, or removing volume limits for specific types of services, as well as changing contracting principles (Table 6).

Our findings are consistent with previous literature showing that output-based payment methods (per case and per unit of service) are prevalent across all types of healthcare providers worldwide [14] and specifically in the CEE region [14,15]. This was related to a common trend to strategically move away from paying for resources (input-oriented methods) to paying for outputs, which could better reflect the actual costs of services [14]. Although output-based methods prevail, some of the analysed countries have quite recently reintroduced input-based payment in the form of fixed budget for salaries. In most cases it was related to the growing pressure of health workforce deficit and the need to secure continuous health services provision. Our results show that outcome-based payment models are rarely used in practice (mainly as single indicators in P4P programs in PHC). This is also in line with the findings of previous studies, which claim that, although outcome-based payment models have gained traction in recent years, they are used with limited scope [40,41], often narrowly focusing on specific diseases or conditions [40,42,43]. This is usually preferred when clear and unambiguous indicators are available to avoid penalizing providers based on (outcome) indicators beyond their control. The latter has been shown to be one of the most common reasons why healthcare providers resist such payment methods, although a number of other barriers, such as the complexity of measurements, data availability and infrastructure, and lack of standardization, pose additional challenges [[42], [43], [44]].

Owing to different payment methods providing different set of incentives for providers, policymakers strive to balance diverse, often conflicting incentives within the provider payment schemes, to achieve some pre-defined health system goals. Our study found that, CEE countries follow international trends by using mixed health care provider payment methods. They often combine input-based fixed payments with a variety of output-based methods and usually single outcome (health) indicators within P4P programs. The existing literature indicates that blended payment methods are necessary to optimally balance the multiple objectives of “pure" provider payment methods such as cost and quality [15,[45], [46], [47], [48]]. Evidently, not all provider payment methods are combinable. For instance, when output-based payment (e.g. capitation, per case) is combined with a line-item budget, there may be conflicting incentives. The provider might want to use staff more efficiently under capitation (or case-based), but also want to scale up the number of higher-paid staff to get a higher supplemental pay (i.e. salary allowance) in their budget [14]. Studies provide evidence on better ways for blending provider payment methods [14,16,46,49]. Consistent incentives are crucial both within individual payment scheme and in the relationships between all payment schemes in use [14,46]. Typical examples in the literature include capitation payments for PHC plus FFS for priority interventions. For episodic care, for example, FFS is used in combination with P4P, and DRGs are used in combination with global budget [50]. Finally, bundled payments are used to keep track of the interface and continuity of care (i.e., the continuum between primary, secondary, and/or tertiary care), especially for continuous and coordinated care for chronic conditions [46,50]. While using blended payment methods for health care provision is a common practice, it also contributes to significant administrative and evaluation challenges (e.g. assessing the impact of a given payment method while controlling for the incentives driven by other methods) [49].

Our results indicate that P4P has been adopted in practically every country, particularly in PHC. While these programs focus on similar areas (e.g. supporting preventative interventions, cancer screening, and chronic illness surveillance) and most often apply output based indicators, the choice of specific objectives, metrics (and/or their thresholds) as well as the form of financial incentive varies between countries. This is in line with previous studies indicating that P4P programs in PHC often focus on process-of-care dimension, yet can be very heterogeneous in terms of specific priorities, indicator choices, and financial reward design [51]. Although in the case of our group of countries the evaluations of P4P programs are scarce, the available international evidence points towards their limited effectiveness in achieving the defined objectives, especially in the long term [43,[51], [52], [53], [54]]. The design of P4P programs and administrative capacity in the health care system play a crucial role in the success of these reforms [53,54]. The choice of monitored indicators should allow for objective and comprehensive performance measurement (that would for example not discriminate against specific providers) while the financial incentive should provide adequate motivation (too low would not motivate desired behaviour, while too high might lead to wasteful spending). Finally, the administrative burden of the program must also be taken into consideration [53]. In consequence, decision-makers should regularly evaluate P4P programs, monitor providers’ behaviours and introduce adequate revisions, optimally agreed in cooperation with providers.

For a given payment method to provide the expected incentives, service tariffication/pricing must be correct (reflecting actual costs) [20]. Our study indicated that modification of tariffs/price-setting principles to better reflect actual cost was common across all provider types as well as all types of payment methods. These processes require i.a. access to a reliable data infrastructure, adequate institutional capacities, and rigorous and transparent methodologies [20]. CEE countries follow international trends in this field [55] by launching dedicated research projects, and pilot studies supervised by dedicated institutions. At the same time, although the trends are similar, countries can choose diverse approaches and be at different levels of advancement. For example, in Poland, the dedicated cost accounting standards for hospitals providing services under contract with public payer were adopted by the government in 2021 [56] while in Estonia, the first initiatives on activity-based costing have been implemented by hospital associations almost two decades ago. This is in line with existing literature indicating that the regulatory frameworks for health services price-setting can vary significantly between countries, as well as within the same country for different types of providers or payers [20]. Available international evidence also indicates that while initiating reforms aimed at improving the price setting process, the policymakers should carefully balance the trade-off between the cost-data accuracy and the available infrastructure/system feasibility constraints [57].

Payment schemes are used to influence provider behavior towards the realization of predefined health policy objectives [14]. Our study showed that, although policymakers across the nine CEE countries might have introduced different changes to the payment methods, the general objectives of these changes were often similar. Efforts have focused i.a. on strengthening PHC provision by supporting disease prevention activities (via dedicated P4P programs and/or expanding the scope of FFS), shifting emphasis from inpatient to outpatient care (by expanding the scope of services financed via FFS, case payments and modifying their tariffication process, modifying or introducing global budgets, adding P4P) and implementing coordinated care models (via adding P4P, FFS or bundled payments). These findings are in line with previous studies on general trends of health reforms in CEE countries [6,7,12]. Also, although our study focused mostly on changes in payment methods, we have also mapped complementary, additional changes in payment system. These focused on volume limits and contracting principles (e.g. extending the contact time from for hospital providers). Volume limit changes could have provided an additional set of incentives to steer provider behavior. For example, in Poland removing volume limits for day care procedures in connection with increasing tariff valuation for these procedures led to significant shift in the procedures settings (e.g. the share of cataract surgeries performed in day care settings in total number of these procedures increased from 24.5 % in 2011 to 93.6 % in 2021 [9]). The policy objective of the reform – shortening waiting time and limiting inpatient care for defined procedures was achieved, yet in the long run the payer was unable to secure regular reimbursements (the payments for unlimited services were delayed) [58]. This strongly impacted providers’ financial security and limited their willingness to offer outpatient procedures. The Polish example emphasises the importance of properly planning payment reforms (i.e. securing payer's budget), monitoring and conducting adequate adjustments.

Our study has several limitations. First is the potential influence of arbitrary and/or subjective factors while updating the data collection forms by the national experts. This risk was mitigated by referring to relevant literature whenever available, but it could not be entirely eliminated. Second, our study was limited to mapping changes in provider payment schemes since 2010. Consequently, we excluded similar payment reforms that might have taken place in some countries prior to 2010 (e.g., introduction of P4P in primary care in Hungary and DRGs in hospital care in Poland). We have only covered the public health system and payment schemes between public payers and providers’ institutions (legal entities) and thus, excluding issues related e.g. to payments within voluntary health insurance systems as well as direct salary payments for medical staff (relations between health care institutions and their employees). This limitation indicates that we have only analysed part of the picture in terms of the interplay between economic incentives provided by different payment methods (i.a. those used by public payers vs. those used by health care institutions to pay their medical staff vs those used in private sector). We have also excluded issues related to payments for pharmaceuticals, medical products, and dental services, which could have shown an even more complex picture of the existing payment methods mix. Finally, although during the data validations process, we aimed at identifying literature references related to specific reforms the evaluation studies were scarce. As a consequence, we have provided mostly narrative descriptions of the changes, without details of their impact assessment.

Regardless of these limitations, our study constitutes a unique, structured comparison of provider payment schemes in public health system across nine CEE countries and provides implications for both policy and research. Health policymakers in CEE countries can learn by comparing international experience. However, the prerequisite is the availability of specific reform evaluations, including reforms impact assessment studies as well as analysis of factors that influenced the success or failure of a given reform. This drives strong implications for future research. Although evaluations of payment reform effectiveness (especially blended payment schemes) are extremely difficult to conduct [45,46,49], they constitute the basis of evidence-informed health policy processes. A description of the evaluation process can be included as part of the reform plan. Regardless of whether it is designed prior to or after reform implementation, the evaluation must be based on a rigorous methodological approach, including the assurance of appropriate data availability and an adequate timeframe for the impact assessment. Research-based knowledge on barriers and facilitators of specific payment reforms can also help in planning adequate mitigating or supporting actions [44,59]. While our study covered only part of the existing health care providers payment schemes, future studies could focus on the interplay between economic motivations of specific payment methods for providers at two levels (organizations and individual health workers) as well as those between public and private payers. Future collaborative, cross-country studies can concentrate on the identified similar reform trends e.g. economic incentives to enhance disease prevention in PHC; reforms aimed at shifting from in- to outpatient care provision or best practices in developing activity-based costing standards for the tariffication purposes.

## 5 Conclusions

The nine Central and Eastern European countries, Bulgaria, Croatia, Czechia, Estonia, Latvia, Lithuania, Hungary, Poland, and Romania, show numerous similarities in terms of the current healthcare provider payment methods mix as well as the general objectives of the changes conducted in this field since 2010. Output-based payment methods prevail across all countries and types of providers. Primary health care providers are characterized by the most diverse payment method mix. PHC and hospital inpatient care have experienced the most frequent changes in their payment schemes within the last 13 years. Although the changes varied across countries, the general objectives of conducted reforms were often similar. They focused on: improving tariffication processes for better reflection of actual costs; expanding the scope of PHC services (with focus on disease prevention, care coordination, and multidisciplinary approach); shifting emphasis from in-patient to outpatient care (for both hospitals and LTC institutions), improved efficiency of hospital care. There is a huge potential for shared learning between CEE countries in terms of provider payment scheme reforms, yet the prerequisite is the availability of robust evaluation studies.

## CRediT authorship contribution statement

**Costase Ndayishimiye:** Writing – review & editing, Writing – original draft, Validation, Methodology, Investigation, Formal analysis, Data curation, Conceptualization. **Marzena Tambor:** Writing – review & editing, Supervision, Methodology, Data curation, Conceptualization. **Daiga Behmane:** Writing – review & editing, Validation, Data curation. **Antoniya Dimova:** Writing – review & editing, Validation, Data curation. **Alina Dūdele:** Writing – review & editing, Validation, Data curation. **Aleksandar Džakula:** Writing – review & editing, Validation, Data curation. **Barbora Erasti:** Writing – review & editing, Validation, Data curation. **Péter Gaál:** Writing – review & editing, Validation, Data curation. **Triin Habicht:** Writing – review & editing, Validation, Data curation. **Pavel Hroboň:** Writing – review & editing, Validation, Data curation. **Liubove Murauskienė:** Writing – review & editing, Validation, Data curation. **Tamás Palicz:** Writing – review & editing, Validation, Data curation. **Silvia Gabriela Scîntee:** Writing – review & editing, Validation, Data curation. **Lenka Šlegerová:** Writing – review & editing, Validation, Data curation. **Cristian Vladescu:** Writing – review & editing, Validation, Data curation. **Katarzyna Dubas-Jakóbczyk:** Writing – review & editing, Writing – original draft, Validation, Supervision, Project administration, Methodology, Investigation, Formal analysis, Data curation, Conceptualization.

## Declaration of competing interest

The authors declare no conflict of interest.

## Appendix Supplementary materials

Image, application 1

## References

[1] Rechel B., McKee M. (2009). Health reform in central and eastern Europe and the former Soviet Union. Lancet.

[2] Romaniuk P., Szromek A.R. (2016). The evolution of the health system outcomes in Central and Eastern Europe and their association with social, economic and political factors: an analysis of 25 years of transition. BMC Health Serv Res.

[3] Tambor M., Klich J., Domagała A. (2021). Financing healthcare in central and eastern European countries: how far are we from universal health coverage?. Int J Environ Res Public Health.

[4] Kutzin J., Jakab M., Cashin C. (2010). Lessons from health financing reform in central and eastern Europe and the former Soviet Union. Heal Econ Policy Law.

[5] Healy J., McKee M. (2002). Implementing hospital reform in central and eastern Europe. Health Policy (New York).

[6] Semánová C. (2019). Primary care behind the former ‘Iron Curtain’: changes and development of primary healthcare provision in the Eastern part of the European Union. Prim Health Care Res Dev.

[7] Dubas-Jakóbczyk K. (2020). Hospital reforms in 11 Central and Eastern European countries between 2008 and 2019: a comparative analysis. Health Policy (New York).

[8] Csanádi M. (2022). Prioritization of implementation barriers related to integrated care models in Central and Eastern European countries. Health Policy (New York).

[9] Eurostat, “Eurostat Database,” 2024. https://ec.europa.eu/eurostat/data/database (accessed Sep. 11, 2024).

[10] K. Hirose and Z. Czepulis-Rutkowska, “Challenges in long-term care of the elderly in Central and Eastern Europe,” Geneva, Switzerland, 2016.

[11] Sowa P.M. (2016).

[12] Sagan A., Kowalska-Bobko I., Bryndová L., Smatana M., Chaklosh I., Gaál P. (2023). What is being done to respond to the rise of chronic diseases and multi-morbidity in Czechia, Hungary, Poland, and Slovakia?. Front Public Heal.

[13] Langenbrunner J., Cashin C., O'Dougherty S. (2009).

[14] Cashin C. (2015).

[15] Kutzin J. (2001). A descriptive framework for country-level analysis of health care financing arrangements. Health Policy (New York).

[16] OECD (2016).

[17] Callenbach M.H.E., Ádám L., Vreman R.A., Németh B., Kaló Z., Goettsch W.G. (2023). Reimbursement and payment models in Central and Eastern european as well as Middle Eastern countries: a survey of their current use and future outlook. Drug Discov Today.

[18] Moreno-Serra R., Wagstaff A. (2010). System-wide impacts of hospital payment reforms: evidence from Central and Eastern Europe and Central Asia. J Health Econ.

[19] Waitzberg R. (2022). Balancing financial incentives during COVID-19: a comparison of provider payment adjustments across 20 countries. Health Policy (New York).

[20] Barber S.L., Lorenzoni L., Ong P. (2019).

[21] European Observatory on Health Systems and Policies, “Health system reviews (HiT series),” 2023. https://eurohealthobservatory.who.int/publications/health-systems-reviews (accessed May 25, 2023).

[22] European Observatory on Health Systems and Policies, “Health Systems and Policy monitor (HSPM),” 2023. https://eurohealthobservatory.who.int/monitors/health-systems-monitor (accessed May 25, 2023).

[23] European Observatory on Health Systems and Policies, “Country health profiles - State of health in the EU,” 2023. https://eurohealthobservatory.who.int/publications/country-health-profiles (accessed May 25, 2023).

[24] European Observatory on Health Systems and Policies, “The HSPM network,” 2023. https://eurohealthobservatory.who.int/monitors/health-systems-monitor/network (accessed May 25, 2023).

[25] Ministry of Health, *Vyhláška č. 315/2022 Sb. o stanovení hodnot bodu, výše úhrad za hrazené služby a regulačních omezení pro rok 2023 [Decree No. 315/2022 Coll. on the determination of point values, the amount of reimbursement for paid services and regulatory restrictions]*. Czech Republic, 2022.

[26] Bryndová L., Šlegerová L., Votápková J., Hroboň P., Shuftan N., Spranger A. (2023). Czechia: health system review. Health Syst Transit.

[27] Dimova A., Rohova M. (2023). Changes in GPs payment target wider population coverage for prophylactic examinations. HSPM Policy analysis.

[28] European Observatory on Health Systems and Policies, “Croatia health Systems in transition (HiT) profile,” *HSPM Country Updates 20 March 2023*, 2022. https://eurohealthobservatory.who.int/monitors/health-systems-monitor/countries-hspm/hspm/croatia-2022/principal-health-reforms/analysis-of-recent-reforms/(accessed May 25, 2023).

[29] A. Dimova et al., “Bulgaria: health system summary,” WHO Regional Office for Europe on behalf of the European Observatory on Health Systems and Policies, Copenhagen, 2022.

[30] Gaal P., Velkey Z., Szerencses V., Webb E. (2021). The 2020 reform of the employment status of Hungarian health workers: will it eliminate informal payments and separate the public and private sectors from each other?. Health Policy (New York).

[31] Kotherová Z., Caithamlová M., Nemec J., Dolejšová K. (2021). The use of diagnosis-related group-based reimbursement in the Czech Hospital care system. Int J Environ Res Public Health.

[32] Sagan A., Rogala M., Buszman P.P., Kowalska-Bobko I. (2021). Improved coordination of care after acute myocardial infarction in Poland since 2017: promising early results. Health Policy (New York).

[33] Basińska E. (2023). Program KOS-Zawał wymaga odświeżenia [Coordinate Care program for Stroke Patients – new adjustments needed]. Polityka Zdrowotna.

[34] K. Kasekamp, “New payment model for stroke treatment,” *HSPM Country Updates 23 November 2020*. https://eurohealthobservatory.who.int/monitors/health-systems-monitor/analyses/hspm/estonia-2018/new-payment-model-for-stroke-treatment (accessed Nov. 19, 2023).

[35] S.G. Scintee and C. Vladescu, “Improving Romania's DRGs system,” *HSPM Country Updates 30 April 2020*, 2020. https://eurohealthobservatory.who.int/monitors/health-systems-monitor/updates/hspm/romania-2016/improving-romania-s-drg-system (accessed May 25, 2023).

[36] AOTMiT - agency for health technology assessment and tariff system 2023, “Tariff system description.” https://www.aotm.gov.pl/en/tariff-system/(accessed Nov. 19, 2023).

[37] Kancelaria Sejmu, *Ustawa 2017 Ustawa z dnia 8 czerwca 2017 r. o sposobie ustalania najniższego wynagrodzenia zasadniczego pracowników wykonujących zawody medyczne zatrudnionych w podmiotach leczniczych (Dz.U.2017poz. 1473) [Law on minimal wages for helathcare profession]*. Poland, 2017.

[38] Dubas-Jakóbczyk K., Kowalska-Bobko I., Sowada C. (2019). The 2017 reform of the hospital sector in Poland – The challenge of consistent design. Health Policy (New York).

[39] M. Rohova, “Disputed budget of the NHIF for 2018,” *HSPM Country Updates 20 December 2017*. https://eurohealthobservatory.who.int/monitors/health-systems-monitor/updates/hspm/bulgaria-2018/disputed-budget-of-the-nhif-for-2018 (accessed May 25, 2023).

[40] Vlaanderen F.P. (2019). Design and effects of outcome-based payment models in healthcare: a systematic review. Eur J Heal Econ.

[41] Michelsen S., Nachi S., Van Dyck W., Simoens S., Huys I. (2020). Barriers and opportunities for implementation of outcome-based spread payments for high-cost, one-shot curative therapies. Front Pharmacol.

[42] Leao D.L.L., Cremers H.-P., van Veghel D., Pavlova M., Hafkamp F.J., Groot W.N.J. (2023). Facilitating and inhibiting factors in the design, implementation, and applicability of value-based payment models: a systematic literature review. Med Care Res Rev.

[43] National Audit Office, “Outcome-based payments schemes: government's use of payment by results,” London, 2015.

[44] Ndayishimiye C., Tambor M., Dubas-Jakóbczyk K. (2023). Barriers and facilitators to health-care provider payment reform – A scoping literature review. Risk Manag Healthc Policy.

[45] Feldhaus I., Mathauer I. (2018). Effects of mixed provider payment systems and aligned cost sharing practices on expenditure growth management, efficiency, and equity: a structured review of the literature. BMC Health Serv Res.

[46] I. Mathauer, “Mixed provider payment systems: what are the issues?,” Geneva, 2017.

[47] R. Messerle and J. Schreyögg, “System-wide effects of hospital payment scheme reforms: the German introduction of diagnosis-related groups,” HCHE Research Paper, Hamburg, 2022.

[48] Quentin W. (2022). How Denmark, England, Estonia, France, Germany, and the USA pay for variable, specialized and low volume care: a cross-country comparison of In-patient payment systems. Int J Heal Policy Manag.

[49] Mathauer I., Dkhimi F. (2019).

[50] Milstein R., Schreyögg J. (2024). The end of an era? Activity-based funding based on diagnosis-related groups: a review of payment reforms in the inpatient sector in 10 high-income countries. Health Policy (New York).

[51] Eckhardt H., Smith P., Quentin W., Busse R, Klazinga N, Panteli D, Quentin W (2019). Improving healthcare quality in europe: characteristics, effectiveness and implementation of different strategies.

[52] Milstein R., Schreyoegg J. (2016). Pay for performance in the inpatient sector: a review of 34 P4P programs in 14 OECD countries. Health Policy (New York).

[53] Papanicolas I., Milstein R., Ryan A.M., Figueroa J.F. (2024). Are alternative payment models the answer to the failures of pay-for-performance?. BMJ.

[54] Eijkenaar F., Emmert M., Scheppach M., Schöffski O. (2013). Effects of pay for performance in health care: a systematic review of systematic reviews. Health Policy (New York).

[55] Barber S.L., Lorenzoni L., Ong P. (2020). Institutions for health care price setting and regulation: a comparative review of eight settings. Int J Health Plann Manage.

[56] Minister of Health, *Rozporządzenie MINISTRA ZDROWIA z dnia 26 października 2020 r. w sprawie zaleceń dotyczących standardu rachunku kosztów u świadczeniodawców [Minister of Health2022, Regulation on cost accounting standard for health care providers]*. Poland, 2022.

[57] Tan S.S. (2014). DRG systems in Europe: variations in cost accounting systems among 12 countries. Eur J Public Health.

[58] Puls Medycyny, “NFZ nie ma pieniędzy na zapłatę za świadczenia nielimitowane? "to problem do rozstrzygnięcia wspólnie z MZ,” 2024. https://pulsmedycyny.pl/nfz-nie-ma-pieniedzy-na-zaplate-za-swiadczenia-nielimitowane-to-problem-do-rozstrzygniecia-wspolnie-z-mz-1217476 (accessed Sep. 20, 2024).

[59] de Vries E.F., Drewes H.W., Struijs J.N., Heijink R., Baan C.A. (2019). Barriers to payment reform: experiences from nine Dutch population health management sites. Health Policy (New York).

