# Supplementary material for: Health care provider payment schemes and their changes since 2010 across nine Central and Eastern European countries – a comparative analysis
Source: Health Policy. 2025 Mar;153:105261. doi: 10.1016/j.healthpol.2025.105261 (PMC11878279; doi:10.1016/j.healthpol.2025.105261)
Supplement: Supplementary file 1 [file mmc1.docx]

**SUPPLEMENTARY TABLES**

**Supplementary Table S1. Payment methods classification overview* – per country and provider type**

| **Type of care & provider / Country** | **PRIMARY HEALTH CARE** | **OUTPATIENT SPECIALIZED CARE (outside hospitals)** | **HOSPITALS** | | **LONG TERM CARE (within health sector)** | |
| --- | --- | --- | --- | --- | --- | --- |
|  |  |  | **Outpatient specialized care (inside hospitals)** | **Inpatient hospital care** | **Inpatient LTC** | **Outpatient/day/ home LTC** |
| **Bulgaria** | Capitation + FFS, P4P | FFS | Case payment | Case payment | Case payments (for acute episodes),  Per diem (for psychiatric care) | Not covered |
| **Croatia** | Capitation + FFS, P4P, Fixed budget, | FFS | FFS, Case payments | Global budget, DRGs, Case payment | Per diem | FFS |
| **Czechia** | Capitation + FFS, P4P, Fixed payments (for emergency care shifts) | FFS + Case payments (day surgeries), P4P elements (dialysis providers) | FFS + Case payments (day surgeries), P4P elements (dialysis providers) | Global budget (based on DRGs), DRGs, Fixed payment (for palliative care) | Per diem | FFS |
| **Estonia** | Capitation, FFS, P4P, Fixed payment (e.g. for distance, second nurse) | FFS + P4P elements (video consultations) | FFS + P4P elements (video consultations) | DRGs, FFS, Per diem, Fixed payment, Bundled payments (for stroke patients) | Per diem, FFS | FFS |
| **Hungary** | Capitation, Case payment, P4P, Fixed payment (for group practices formulation and salaries) | FFS + Fixed payment (for salaries) | FFS + Fixed payment (for salaries) | DRGs + Fixed payment (for salaries) | Per diem + Fixed payment (for salaries) | Per diem (hospice home care), Per visit (specialist home care) |
| **Latvia** | Capitation, FFS, Fixed payment (for salaries), P4P | FFS, Case payment, Fixed payment (for salaries) | FFS, Case payment | DRGs, Case payment, Per diem, Fixed payment (for emergency care), FFS | Fixed budget, Per diem | Case payment, FFS, |
| **Lithuania** | Capitation, FFS, P4P, Fixed payment (for special needs patients) | Case payment | Case payment, FFS (for expensive procedures and examinations) | DRGs, FFS | Per diem, Case payment (palliative care) | Per diem, FFS, Fixed payment (nursing at home), Case payment (palliative care) |
| **Poland** | Capitation + Per visit/consultation, FFS (for diagnostic tests and within coordinated care), Fixed payment (for rural/low density population) | Per visit payment (groups adjusted for number and type of services provided) + FFS | Per visit payments (groups adjusted for number and type of services provided) + FFS, P4P elements (oncological network) | Global budget (based on DRGs) for hospital included in network + DRGs, P4P elements (for stroke patients), FFS, per diem | Per diem (differentiated based on health and care needs) | Per diem (differentiated based on health and care needs) |
| **Romania** | FFS + Capitation, P4P, Fixed payment(for newcomers) | FFS | FFS | DRGs + Case payment, Fixed payment (for salaries) | Per diem | FFS, Case payment |

*payment method category is marked by colors: input based; output based – per capita; output based - per case; output based - per unit of service; outcome based (see Table 1 in the main manuscript); while terms case-payment and DRGs are used in the literature as synonyms, the distinction between the two have been made based on the cost groups complexity level with DRGs being a more complex method (cost groups based on set of characteristics including three dimensions: diagnosis, procedures and patient’s features) while case payment can take a simpler form (e.g. series of procedures for the same diagnosis, without patients characteristics for the day surgeries in Czechia; care provided by outpatient specialist during 30 days in Latvia; or clinical pathways in hospitals and LTC in Bulgaria)

**Supplementary Table S2. Overview of main changes to payment methods* and their motivations**, per country and provider type, since 2010**

| **Type of care & provider / Country** | **PRIMARY HEALTH CARE** | **OUTPATIENT SPECIALIZED CARE (outside hospitals)** | **HOSPITALS** | | **LONG TERM CARE (within health sector)** | |
| --- | --- | --- | --- | --- | --- | --- |
|  |  |  | **Outpatient specialized care (inside hospitals)** | **Inpatient hospital care** | **Inpatient LTC** | **Outpatient/day/ home LTC** |
| **Bulgaria** | **‘MOD’** (ongoing: new capitation tariffs, age adjusted FFS, FFS tariff modification aimed at better reflection of actual costs)  +  **‘ADD’** (2022: P4P elements for prophylaxis aimed to enhance population coverage with prophylactic services) | **‘MOD’** (new tariffs valuation aimed at better reflection of actual costs) | **‘MOD’** (2016: new ambulatory procedures to improve efficiency, new tariffs aimed at better reflection of actual costs) | **‘MOD’** (increased number and tariffs of clinical pathways to better reflect the actual costs) | ‘NO CHANGES’ | ‘NO CHANGES’ |
| **Croatia** | **‘ADD’** (2013: P4P to encourage provision of certain types of care (e.g. preventive care) and to improve quality of care and patient satisfaction) | ‘NO CHANGES’ | **‘MOD’** (2015: introducing diagnostic procedures to encourage more services being provided in out-patient settings) | **‘MOD’** (2015: refined DRGs to make payment more transparent and related to actual costs, to encourage more effective use of resources) | ‘NO CHANGES’ | ‘NO CHANGES’ |
| **Czechia** | **‘MOD’** (ongoing: increasing capitation, expanding the scope of services financed via FFS aimed at strengthening the intensity and scope of PHC services )  +  **‘ADD’** (2016: fixed bonus/payment for emergency care shifts – to encourage PHC doctors to work in emergency care, to fill-in the physicians deficit) | ‘**MOD**’ (ongoing: new FFS tariffs, expanding the scope of services financed via FFS to encourage more services being provided in out-patient settings )  +  **‘ADD’** (2019-2020: P4P for dialyses providers to encourage better access and quality of care, 2023: case payment for day surgery to encourage more procedures being performed in day settings) | **‘MOD’** (ongoing: changes in reimbursement formula, new FFS tariffs, expanding the scope of services financed via FFS to encourage more services being provided in out-patient settings)  +  **‘ADD’** (2019-2020: P4P for dialyses providers to encourage better access and quality of care, 2023: case payment for day surgery to encourage more procedures being performed in day settings) | **‘MOD’** (2012: activity based global budgets aimed at better flexibility of services provided within hospital budgets, 2019: DRGs modifications to make costing group more detailed and better reflect the actual costs)  +  **‘ADD’** (2023: fixed payment based on number of insured for palliative care to encourage provision of palliative services)  +  **‘REP’** (2021: CZ-DRGs aimed at better reflection of actual costs) | **‘MOD’** (2016: ongoing tariffs differentiation, new reimbursement rules aimed at better reflection of actual costs) | **‘MOD’** (2018-2019: expanding scope of services financed via FFS to encourage more LTC services to be provided in home settings) |
| **Estonia** | **‘MOD’** (2012: new capitation groups, ongoing: tariff valuation changes to make costing group more detailed and better reflect the actual costs)  +  **‘ADD’** (2013: FFS for e-consultations to encourage more services to be provided remotely; 2015: P4P to encourage better care coordination especially for chronic disease and strengthen health promotion activities, 2017: fixed payment to encourage setting up group practices) | **‘ADD’** (2021: video consultations P4P to encourage more services to be provided remotely) | **‘ADD’** (2021: video consultations P4P to encourage more services to be provided remotely) | **‘ADD’** (2020: fixed payment for emergency care to secure services provision; 2021: bundled payment for stroke patients for better care coordination) | **‘MOD’** (ongoing tariffs differentiation aimed at better reflection of actual costs) | **‘MOD’** (ongoing tariffs differentiation aimed at better reflection of actual costs) |
| **Hungary** | **‘ADD’** (2021: Fixed payment to encourage group practices, 2021: fixed payment for salaries to cover regulatory wages increase) | **‘ADD’**(2021: fixed payment for salaries to cover regulatory wages increase) | **‘ADD’** (2021: fixed payment for salaries to cover regulatory wages increase) | **‘MOD’** (ongoing tariffs adjustments aimed at better reflection of actual costs)  +  **‘ADD’** (2021: fixed payment for salaries to cover regulatory wages increase) | **‘ADD’**(2021: fixed payment for salaries to cover regulatory wages increase) | ‘NO CHANGES’ |
| **Latvia** | **‘ADD’** (2013: FFS to encourage more services being provided in PHC and P4P to enhance health promotion and disease prevention activities) | **‘MOD’** (ongoing tariffs adjustments aimed at better reflection of actual costs) | **‘MOD’** (ongoing tariffs adjustments aimed at better reflection of actual costs) | **‘REP’** (2011: DRGs DRGs to make payment more transparent and related to actual costs, to encourage more effective use of resources)  +  **‘MOD’** (DRGs modifications to make costing group more detailed and better reflect the actual costs) | **‘MOD’** (ongoing: changes in the scope of services financed via given method to incentivize provision of specified services) | **‘MOD’** (ongoing: changes in the scope of services financed via given method to incentivize provision of specified services) |
| **Lithuania** | **‘MOD’** (ongoing tariffs differentiation, expanding the scope of services financed via FFS and P4P aimed at strengthening PHC services intensity and scope by e.g. adding more diagnostic tests, health prevention activities to be carried by primary care doctors and involving supporting health workers ) | **‘MOD’**(new tariffs, expanding the list of services financed via case payments; 2016: extended consultations to encourage more services being provided in out-patient settings) | **‘MOD’**(new tariffs, expanding the list of services financed via given methods; 2016: extended consultations to encourage more services being provided in out-patient settings) | **‘REP’** (2012: DRGs to make payment more transparent and related to actual costs, to encourage more effective use of resources, to allow hospital comparisons) + **‘MOD’** (2015: country specific DRGs weights aimed at better reflection of actual costs) | **‘MOD’** (2018-2022: ongoing tariffs differentiation aimed at better reflection of actual costs) | **‘MOD’** (2019: new tariffs for palliative care to encourage more LTC services to be provided in out-, home and/or day settings) |
| **Poland** | **‘MOD’** (adjusted capitation groups, expanding scope of services financed via FFS aimed at strengthening PHC services intensity and scope by e.g. adding more diagnostic tests, health promotion activities to be carried by primary care doctors)  +  **‘ADD’** (2019: fixed payment for rural areas aimed at encouraging setting practices in rural areas) | **‘REP’** (2011: per visit adjusted for number and type of services provided aimed at better reflection of actual costs)  +  **’MOD’** (new tariff valuation rules, new rules for reimbursement calculation aimed at better reflection of actual costs and to encourage more services being provided in out-patient settings) | **‘REP’** (2011: per visit adjusted for number and type of services provided aimed at better reflection of actual costs)  +  **‘MOD’**(new tariff valuation rules, new rules for reimbursement calculation to encourage more services being provided in out-patient settings)  +  **‘ADD’**(2017-2022: global budget aimed at shifting emphasis from in- to out-patient services within hospital budget, 2015: P4P elements within oncological network, FFS and per diem for oncological coordinated care aimed at improving access to oncological diagnostic and treatment) | **‘MOD’** (new tariff valuation rules, new rules for reimbursement calculation aimed at better reflection of actual costs)  +  **‘ADD’** (2017: global budget aimed at better flexibility of services provided within hospital budgets and shifting emphasis from in- to -outpatient care; 2015: P4P elements within oncological pathways, FFS and per diem for oncological coordinated care aimed at improving access and coordination of oncological diagnostic and treatment, 2022: P4P elements within stroke program aimed at better access and coordination of care for stroke patients) | **‘MOD’**(2015: gradual tariffication of services, differentiation of per diem payment depending on health needs aimed at better reflection of actual costs) | **‘MOD’**(2015: gradual tariffication of services; differentiation of per diem depending on health needs aimed at better reflection of actual costs) |
| **Romania** | **‘MOD’** (expanding the scope of services financed via FFS to encourage more services being provided in PHC, e.g. preventive services more diagnostic test) +  **‘ADD’** (2023: P4P to encourage provision of preventive services, e.g. health risk assessment) | **‘MOD’** (ongoing changes to the tariff valuation rules to make costing group more detailed and better reflect the actual costs) | **‘MOD’** (extending the list of services financed via FFS to encourage more services being provided in out-patient settings, ongoing changes to the tariff valuation rules aimed at better reflection of actual costs) | **‘MOD’** (new tariff rules for DRGs in Romanian context 2020 aimed at better reflection of actual costs)  +  **‘ADD’** (2017: additional, fixed payment to cover regulatory salaries increase) | ‘NO CHANGES’ | **‘MOD’** (2014: changes to tariffs calculation methods for home care aimed at better reflection of actual costs)  +  **‘ADD’**(2018: payment for outpatient palliative care to encourage more services provision) |

*Major changes to payment methods, including four options: ‘NO CHANGES’ (no change in the payment methods since 2010 as well as no major modification to its content); REP’ – replacing previous method to new one; ‘MOD’ – modifying the existing method (e.g. by changing number of DRGs/capitation groups, modifying costing groups by making them more detailed, changing tariff valuation rules, expanding the scope of services financed via given method); ‘ADD’ – adding new (additional method) to the existing one. ** description of main motivations behind specific changes is marked by red colour
